# Embolie pulmonaire: indice de sévérité de l’embolie pulmonaire (ISEP) score et facteurs prédictifs de mortalité

**DOI:** 10.11604/pamj.2023.45.48.39031

**Published:** 2023-05-19

**Authors:** Majed Hassine, Mohamed Yassine Kallala, Marouen Mahjoub, Mehdi Boussaada, Nidhal Bouchahda, Habib Gamra

**Affiliations:** 1 Cardiology A Department, Fattouma Bourguiba University Hospital, Cardiothrombosis Research Laboratory (LR12SP16), University of Monastir, Monastir, Tunisia

**Keywords:** Embolie pulmonaire, pronostic, stratification du risque, mortalité, Pulmonary embolism, prognosis, risk stratification, mortality

## Abstract

**Introduction:**

l´embolie pulmonaire constitue par sa morbi-mortalité demeurant élevée un véritable problème de santé publique. L´objectif de cette étude est d´évaluer l'impact de l'ISEP (indice de sévérité de l'embolie pulmonaire) score sur le pronostic de l´embolie pulmonaire.

**Méthodes:**

étude rétrospective qui a colligé 146 cas d'embolies confirmées de façon formelle. A partir de l´ISEP score calculé pour l´ensemble de cette population, nous avons subdivisé nos patients en 2 groupes: un groupe à bas risque (BR) regroupant les classes I et II: 83 patients; un groupe à haut risque (HR) regroupant les classes III, IV et V: 63 patients. Le critère de jugement primaire de l´étude (MACE) regroupait la survenue d´un état de choc, la nécessité de ventilation mécanique, et la survenue d'un décès en intra-hospitalier.

**Résultats:**

la mortalité intra-hospitalière totale était de 15,1%, significativement plus importante dans le groupe à HR (25,4% contre 7,2%, p=0,001). En analyse de régression logistique, l'appartenance au groupe HR (OR=5,1; IC à 95%: [1,637 - 16,093]; p=0,005) et l'insuffisance rénale (OR=4,5; IC à 95%: [1,457 - 14,075]; p=0,009) étaient les facteurs indépendants de survenue de MACE. Au bout d´un suivi moyen de 18 ± 8 mois, on a noté plus de décès dans le groupe HR (68,4% contre 33%, p=0,004).

**Conclusion:**

les résultats de notre étude démontrent que l´ISEP score est corrélé à la sévérité de l´EP ce qui devrait encourager la généralisation de l´utilisation de ce score de risque.

English abstract

## Abstract (en)

**Introduction:**

pulmonary embolism is a real public health problem because of its high morbidity and mortality rate. The purpose of this study is to evaluate the impact of PESI score on the prognosis of pulmonary embolism.

**Methods:**

we conducted a retrospective study of 146 cases of formally confirmed embolism. Pulmonary embolism severity index (PESI) score was calculated for the entire study population. The patients were divided into 2 groups: a low-risk group (LR) comprising patients in risk classes I and II: 83 patients; a high-risk group (HR) comprising patients in risk classes III, IV and V: 63 patients; the primary endpoint of the study (MACE) was the occurrence of shock, the need for mechanical ventilation, and the occurrence of in-hospital death.

**Results:**

total in-hospital mortality was 15.1%, significantly higher in the HR group (25.4% versus 7.2%, p=0.001). In logistic regression analysis, being in the HR group (OR=5.1; 95% CI: [1,637 - 16,093]; p=0.005)) and having renal impairment (OR=4.5; 95% CI: [1.457 – 14.075]; p=0.009) were the independent factors for the occurrence of MACE. After a mean follow-up of 18 ± 8 months, there were more deaths in the HR group (68.4% versus 33%, p=0.004).

**Conclusion:**

the results of our study show that the PESI score is correlated with the severity of PE. This should encourage the widespread use of this risk score.

## Introduction

L´embolie pulmonaire (EP) est un problème majeur de santé publique par sa morbi-mortalité et ses répercussions socio-économiques. Elle représente la 3^e^ pathologie cardio-vasculaire en termes d´incidence [1]. L´une des principales difficultés de la prise en charge de l´EP réside dans le diagnostic initial. En effet, la sémiologie de l´EP est peu spécifique et requiert une standardisation par des scores prédictifs de probabilité clinique [2,3].

L´évaluation initiale du patient atteint d´EP repose actuellement sur le niveau de risque de décès précoce [4,5]. L'affinement de la stratification de ce risque a bénéficié récemment de l'avènement de nouveaux scores [1] dont l'ISEP score (indice de sévérité de l'embolie pulmonaire).

Le haut niveau de sensibilité de l´ISEP permet d´identifier efficacement et simplement les malades à très faible risque de décès. Ces scores restent moins validés pour les patients à risque intermédiaire ou élevé pouvant être proposés pour un traitement fibrinolytique [6,7]. Nous nous proposons ainsi comme objectifs: 1) d´identifier les facteurs prédictifs de la mortalité intra-hospitalière et de survenue d´événements cardiovasculaires indésirables majeurs (MACE) causés par l'EP; 2) d´évaluer l'impact pronostic pratique de l'ISEP score.

## Méthodes

**Design de l´étude:** il s'agit d'une étude de cohorte rétrospective mono-centrique, à visée analytique et descriptive. Cette étude a porté sur l´analyse des données de patients admis pour EP aiguë de différents degrés de gravité. Nous nous sommes intéressés au devenir de ces patients en intra-hospitalier et à court et moyen terme.

**Figure 1:**
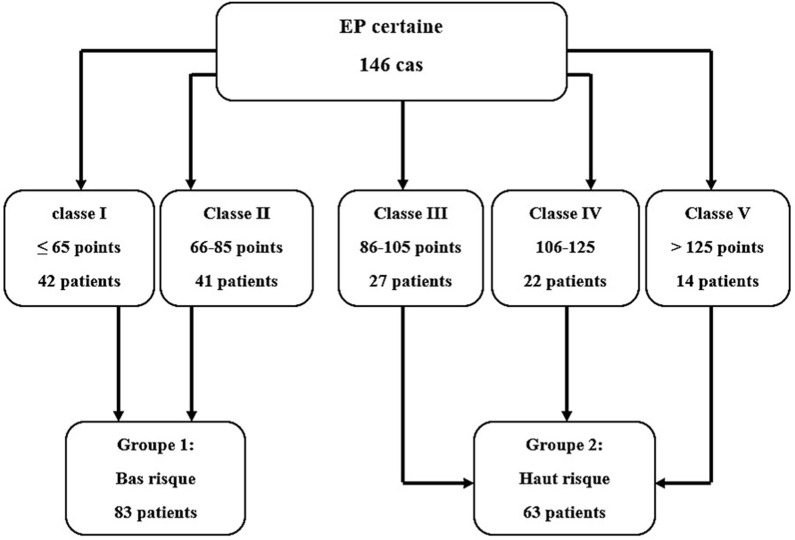
répartition de la population de l´étude

**Cadre de l´étude:** notre étude a été menée au service de cardiologie A du CHU Fattouma Bourguiba de Monastir. Le diagnostic d´embolie pulmonaire aiguë a été retenu de façon formelle soit par un angioscanner pulmonaire multibarette retrouvant un défect endoluminal ou par une scintigraphie pulmonaire de ventilation/perfusion de haute probabilité. Tous les patients éligibles ont été inclus à partir de janvier 2015 jusqu'à décembre 2020 (Figure 1).

**Participants:** les critères d´éligibilité étaient: âge > 18 ans; patient hospitalisé au service de cardiologie A et désireux de participer à l´étude; patients ayant été diagnostiqué formellement porteur d´EP aiguë. Les données relatives à ces patients ont été recueillies à partir de leurs dossiers médicaux. Nous avons exclu les patients ayant une espérance de vie inférieure à 30 jours en rapport avec d´autres pathologies ainsi que les patients perdus de vue. L´ISEP score a été calculé pour chaque patient à partir du moment du diagnostic d´EP. A partir de ces données, nous avons subdivisé les patients en 2 groupes [8,9]: 1) groupe 1: à bas risque (BR) regroupant les classes I et II: 83 patients; 2) groupe 2: à haut risque (HR) regroupant les classes III, IV et V: 63 patients. Une échocardiographie trans-thoracique (ETT) a été effectuée de façon systématique nous permettant d´étudier la fonction biventriculaire.

**Conception du suivi:** le suivi de ces patients a consisté en un examen clinique et un électrocardiogramme (ECG) à 3 mois de la sortie de l´hôpital, puis par intervalles de 6 mois. Le recueil des données du suivi a été réalisé à partir des comptes-rendus de consultation et d'hospitalisation ou par contact téléphonique avec le patient ou son médecin traitant. L´examen clinique était axé sur le dépistage d´une dyspnée ou d´une douleur thoracique résiduelle, de la récurrence d´une maladie veineuse thromboembolique ou de la survenue d´un accident hémorragique. Le critère de jugement primaire de l´étude est un critère composite regroupant la survenue au cours de l'hospitalisation d´un état de choc (EDC), le recours à la ventilation mécanique ou le décès intra-hospitalier. La récidive de l'EP a été attestée par la présence d'un nouveau défect perfusionnel à la scintigraphie pulmonaire ou une nouvelle localisation embolique à l'angio-scanner.

**Analyse statistique:** l´analyse des données a été réalisée par le logiciel SPSS version 23.0 pour Windows. Les variables qualitatives étaient exprimées en termes de fréquences et de pourcentages. Les variables quantitatives étaient exprimées par des moyennes, des médianes plus ou moins des écarts types et dans certains cas nous avons relevé l´étendu (les valeurs minimales et maximales). Pour les critères de jugement primaire, nous avons déterminé l´incidence de survenu durant le premier mois, la première année, puis durant toute la période de suivi. Les comparaisons des moyennes sur des séries indépendantes étaient effectuées au moyen du test t de Student pour les séries indépendantes.

Les comparaisons des pourcentages sur des séries indépendantes étaient effectuées par le test de Chi-deux de Pearson, et en cas de non-validité de ce test nous avons eu recours au test exact bilatéral de Fisher. Une valeur de p inférieure à 0,05 est considérée comme statistiquement significative. L'estimation des données de survie était effectuée par une courbe d´estimation de Kaplan-Meier pour la fonction de survie.

Les comparaisons des fonctions de survie entre les deux groupes étaient effectuées au moyen du test du log rank. Une valeur de p inférieure à 0.05 est considérée comme statistiquement significative. Compte tenu de la nature longitudinale de notre étude, nous avons utilisé la méthode d'analyse des données de survie et le modèle de régression à risque proportionnel de Cox pour établir une relation paramétrique entre les facteurs prédictifs de survenue de l'évènement et la distribution des durées de survie.

## Résultats

**Analyse descriptive non comparative:** cent quarante-six patients ont été inclus au total. La répartition de la population de l´étude selon le niveau de risque est décrite dans la Figure 1. L'âge moyen était de 58.65 ± 18.2 ans. La population de l´étude était composée d´une légère majorité de femmes (52.7%). L'EP était survenue suite à un alitement prolongé postopératoire chez le 25% des patients. Douze patients (8.2%) étaient porteurs de néoplasie. Les principaux signes fonctionnels étaient la dyspnée, la douleur thoracique et l´hémoptysie (Tableau 1). L'électrocardiogramme (ECG) était pathologique chez 126 patients de l'ensemble des patients (86.3%). Une fibrillation auriculaire était présente chez 10 (6.8%) patients. Un aspect S1Q3 était noté chez 33 patients (27%). Un bloc de branche droit (BBD) était noté chez 44.5% des patients. Seuls les patients du groupe HR ont présenté un BBD complet (9.5%). L'ETT a été pratiquée chez les 89.7% des patients et était évocatrice du diagnostic dans 27.4% des cas. Cette présomption était attestée par un septum intra-ventriculaire paradoxal (16.4%), une hypokinésie du ventricule droit (19.2%) et une hypertension pulmonaire avec PAPS ≥ à 40 mmHg (33%). L´angio-scanner a été réalisé pour 90 patients (61.7%) de la population et est revenu normal pour 2 malades appartenant tous les deux au groupe BR dont le diagnostic a été retenue sur les données de la scintigraphie pulmonaire de ventilation et de perfusion. La mortalité intra-hospitalière totale était de 15.1%. Un suivi avec un recul moyen de 18 ± 8 mois a été réalisé auprès de 119 patients (81.5%). Nous avons rapporté 19 cas de décès durant ce suivi.

**Tableau 1:** principaux signes fonctionnels et physiques de l'EP

Signes fonctionnels et physiques	Total N=146	HR N=63	BR N=83	p
Dyspnée, n(%)	120 (82,2%)	59 (93,7%)	61(73,5%)	0,03*
Douleur thoracique, n(%)	81(55,5%)	32 (50,8%)	33 (39,7%)	0,41*
Hémoptysie, n(%)	8 (5,5%)	2 (3,2%)	6 (7,2%)	0,42*
Toux, n(%)	18 (12,3%)	8 (12,7%)	10 (12%)	0,73*
Syncope, n(%)	12(8,2%)	7(11,1%)	5(6%)	0,38*
FC > 100 bpm, n(%)	48 (32,9%)	33(52,4%)	15 (18,1%)	<0,001*
PAS (mm Hg) ± DS, n(%)	121,58 ± 22	125,18 ± 20,2	116,83 ± 23,5	0,023**
Signes d'insuffisance cardiaque droite, n(%)	36 (24,7%)	21(33,3%)	15 (18,1%)	0,06*
Signes de thrombophlébite, n(%)	64(45,8%)	26 (41,3%)	38 (45,8%)	0,39*

EP: embolie pulmonaire; HR: Haut risque; BR: bas risque; FC: fréquence cardiaque; bpm: battements par minute; PAS: pression artérielle systolique; DS: déviation standard; p: groupes comparés: patients à Haut risque vs groupe à bas risque; *: test chi-deux de Pearson ou test exact bilatéral de Fisher comme approprié; **: test t de Student

**Analyse univariée:** il n´y avait pas de différence significative dans la répartition des patients selon le sexe entre les deux groupes (p=0.87). Les patients appartenant au groupe à HR étaient significativement plus âgés (69.7 ± 14.3 ans vs 50.20 ± 16.2; p < 0.001). Le groupe HR comportaient plus de patients porteurs de néoplasie (15.9% vs 2.4%, p=0.012). De tous les signes physiques répertoriés, seule la survenue de la dyspnée se répartissait de manière significativement différente entre les deux groupes avec une nette majorité dans le groupe HR (93.7 % vs 73.5 %, p=0.03). A l´examen physique, les patients du groupe HR avaient une fréquence cardiaque significativement plus élevée (p<0.001) (Tableau 1). Le groupe HR présentait significativement plus d´aspect de BBD (41.3% vs 39.8%, p=0.01). Un aspect S1Q3 était plus fréquemment dans le groupe HR mais sans atteindre le taux de significativité statistique (27% vs 19.3%, p=0.2). Le siège du thrombus était le plus souvent proximal (30.8%) avec répartition égale entre les 2 groupes (31.3% vs 30.2%, p=0.37). L'insuffisance rénale définie par une clairance inférieure à 60 ml/min/1,72 m^2^était plus fréquente chez les patients à haut risque (36.7% contre 12.5%, p=0.001) (Tableau 2).

**Tableau 2:** les principaux paramètres biologiques

Paramètres biologiques	N=146	HR N=63	BR N=83	p
SaO2(%) ± DS	92,9 ± 5,6	90,7 ± 6,85	94,5 ± 3,73	<0,001**
Hémoglobine(g/100ml) ± DS	11,7 ± 2,8	11,4 ± 2,32	11,9 ± 2,29	0,16**
Clairance <60 ml/min, n(%)	32 (22,9%)	22 (36,7%)	10 (12,5%)	<0,001*
D-Dimères (+), n(%)	93 (63,7%)	44 (69,8%)	49 (59%)	0,39*

HR: Haut risque; BR: bas risque; SaO2: saturation artérielle en oxygène; DS: déviation standard; p: groupes comparés: Patients à Haut risque vs groupe à bas risque; *: test Chi-deux de Pearson ou test exact bilatéral de Fisher comme approprié; **: test t de Student

L'évolution intra-hospitalière était dépourvue de complication chez 84.2% des patients et ceci de façon plus significative en cas de BR (90.4% vs 76.2%, p=0.04). Le critère primaire de jugement composite était significativement plus marqué dans le groupe HR (46% vs 9.9%, p<0.0001). La mortalité intra-hospitalière totale était significativement plus importante dans le groupe HR (p=0.001). Le groupe HR comportait significativement plus de cas d´hémorragie non fatale (p=0.02) et de MACE. Le Tableau 3 détaille les complications intra-hospitalières et leurs répartitions. Dix-neuf cas de décès ont été rapportés au cours du suivi et ceci plus fréquemment dans le groupe HR (68.4% vs 33%, p=0.004). Une insuffisance rénale avait également été notée 3 fois plus fréquemment chez les patients décédés (44.4% vs 13%, p=0.001). En analyse univariée, seules l´appartenance au groupe HR (p=0.004) et l´existence d´une insuffisance rénale (p=0.001) ont été prédictifs de mortalité.

**Tableau 3:** les complications survenues en intra-hospitalier

Complications	Total N=146	HR N=63	BR N=83	p
Hémorragie, n(%)	20 (13,7%)	14 (22,3%)	6 (7,2%)	0,02*
Ventilation mécanique, n(%)	14 (9,6%)	10 (15,9%)	4 (4,8%)	0,07*
EDC, n(%)	10 (6,8%)	10 (15,9%)	0 (0%)	<0,0001*
Décès, n(%)	22 (15,1%)	16 (25,4%)	6 (7,2%)	0,001*
MACE	37 (25,7%)	29 (46%)	8 (9,9%)	<0,0001*

HR: Haut risque; BR: bas risque; EDC: état de choc; MACE: évènements indésirable cardiovasculaires majeurs; P: groupes comparés: Patients à Haut Risque vs groupe à bas risque; *: test Chi-deux de Pearson ou test exact bilatéral de Fisher comme approprié

**Figure 2:**
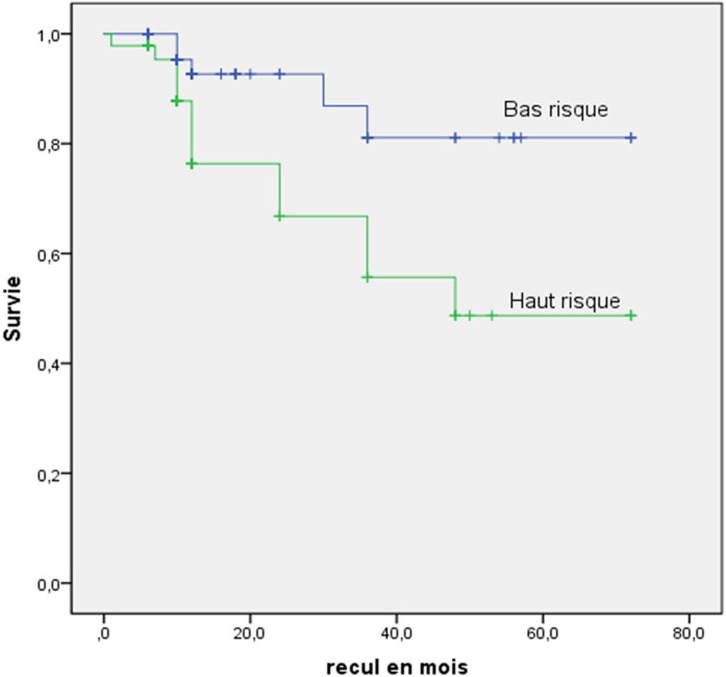
courbe de survie sans MACE

**Analyse multivariée:** la corrélation entre niveau de risque de l´EP et l´insuffisance rénale se confirme en analyse multivariée qui objective que l´appartenance au groupe HR (OR=5.1; IC à 95%: [1.637 - 16.093]; p=0.005) et l´existence d´une insuffisance rénale (OR=4.5; IC à 95%: [1.457 - 14.075]; p=0.009) sont des deux seuls facteurs indépendants de survenue de MACE (Tableau 4). La courbe de survie de Kaplan-Meier sans MACE était significativement différente entre les deux groupes avec une divergence à partir de 10 mois, en faveur du groupe BR (Figure 2).

**Tableau 4:** principaux facteurs prédictifs de MACE en analyse multi-variée

Facteurs prédictifs de MACE	Total (N=146)	Absence de MACE (N=109)	MACE (N=37)	p*
HR , n(%)	63(43,1%)	37 (33,9%)	26(70,2%)	0,005
Tachycardie, n(%)	37(31,3%)	31(32%)	6(27,3%)	0,6
HTAP, n(%)	33(27,7%)	27(27,8%)	6(27,3%)	0,9
Insuffisance rénale, n(%)	32(21,9%)	16(14,6%)	16(43,2%)	0,009
Insuffisance cardiaque, n(%)	14(11,8%)	12(12,4%)	2(9,1%)	0,4

HR: Haut risque; MACE: évènements indésirable cardiovasculaires majeurs; HTAP: hypertension artérielle pulmonaire; p: groupes comparés: Patients n’ayant pas présenté de MACE vs Patient ayant présenté au moins un épisode de MACE; *modèle de régression à risque proportionnel de Cox

## Discussion

La stratification de la sévérité de l´EP est basée sur son retentissement hémodynamique. Ceci est attesté par la clinique, les scores de risque, l´imagerie et la biologie [10-12]. Notre étude s´intègre parmi les différents registres rapportés dans la littérature évaluant l´intérêt des scores de risque. La courbe décrivant la relation entre la sévérité de l´EP rapportée à la mortalité est une courbe exponentielle. La majoration de la sévérité clinique de l´EP au niveau de la partie initiale de la courbe entraine une variation minime de la mortalité. En contrepartie, une variation minime de la sévérité au niveau de la seconde partie de la courbe entraine une élévation majeure du taux de décès. Le point d´inflexion de cette courbe reste sujet à débats [13]. Notre étude permet de mettre l´accent sur les différentes particularités locales de la population et des pratiques médicales.

**Résultats clés et interprétation:** dans notre série le taux global de mortalité, indépendamment de la sévérité était de 15,1%. Les seuls facteurs indépendants prédictifs de survenue de décès intra-hospitalier et de MACE étaient l'insuffisance rénale et l'appartenance au groupe HR. Dans le registre multicentrique *International Cooperative Pulmonary Embolism Registry* (ICOPER), incluant 2454 patients, la mortalité a été de 11,4% à deux semaines, s'élevant à 17,4% à trois mois. Le facteur prédictif indépendant le plus puissant de survenue de décès était la présence d'une PAS < 90 mmHg [14]. Dans notre cohorte, l´hypotension est plus fréquente dans le bras HR sans permettre de prédire la survenue de MACE ou de décès.

La série de Comfere *et al*. comportait un taux de mortalité global de 25,3%. L'instabilité hémodynamique a représenté le paramètre le plus puissant associé à la mortalité. En effet, dans cette série 94.4% des patients ayant nécessité un support vasopresseur à dose importante à l´admission sont décédés [15]. Outre le retentissement hémodynamique, Heit *et al*. ont démontré que le nombre et l´importance des comorbidités représente aussi un facteur de risque majeur de mortalité [16]. D´autres facteurs de risque de mortalité ont été identifiés comme étant l´âge, l´existence d´une insuffisance respiratoire ou cardiaque chronique et la présence d´un cancer [14]. Notre série s´aligne sur les données de littérature en affirmant l´importance de l´instabilité hémodynamique au cours de l´hospitalisation dans la prédiction de la mortalité mais souligne aussi l´insuffisance rénal comme étant un facteur majeur dans la survenue de mortalité intra-hospitalière.

C´est en partant de ces données de littérature que le score ISEP et sa version simplifiée ISEPS ont été validés dans l´évaluation de la sévérité de l´EP en se focalisant uniquement sur les données anamnestiques et cliniques [17-19]. L'ISEP score classe une proportion significativement plus élevée de patients comme étant à BR et a un pouvoir discriminant plus important que l'ISEPS. Notre série confirme le rôle de l´ISEP score dans la prédiction de la mortalité intra-hospitalière. Il a été aussi démontré une corrélation de ce score avec mortalité à 30 et 90 jours et qu´il représente un outil reproductible de stratification du risque d´ EP sévère [8,20]. Ceci s´accorde avec les données de notre cohorte étant donné que durant le suivi, significativement plus de patients à HR sont décédés. Une mortalité plus élevée à 5 ans a été suggérée par Sandal *et al*. chez les patients à HR et rapportée à la présence ou non de comorbidités [21].

Associés à l´ISEP et l´ISEPS scores, le taux de troponine et l´imagerie du ventricule droit permettent d´affiner le niveau de risque et de prévention de la mortalité [1,22]. Ces paramètres permettent ainsi d´identifier les patients à risque intermédiaire haut. L´efficacité de la thrombolyse chez ces patients reste sujette à controverse. En effet, Meyer *et al*. ont démontré l´efficacité de la fibrinolyse dans la prévention de l´instabilité hémodynamique chez ces patients, mais au prix de significativement plus d´évènements hémorragiques majeurs. L´agent fibrinolytique utilisé était exclusivement la ténéctéplase qui ne figure actuellement pas dans les recommandations [1,23,24].

Néanmoins, l´ISEPS identifie avec précision les patients avec EP, qui sont à faible risque de décès [25]. Ainsi, si l´objectif est d´identifier les patients à BR en vue d´un temps de prise en charge plus concis, l'ISEPS est une alternative prometteuse à l'ISEP score original, ce dernier étant plus complexe à calculer [26]. En association avec l´ISEPS les critères Hestia ont été récemment validés afin de permettre de trier les patients à faible risque pouvant être traités à domicile. Ces critères exclus les patients grave, à HR et les patients ayant des facteurs prédictifs de mortalité retrouvés dans notre cohorte comme l´insuffisance rénale sévère [27-29].

**Limites de l´étude:** les principales limites de l´étude sont liées à son caractère rétrospectif et à la taille de l´échantillon étudié. Le caractère rétrospectif du recueil de données a ainsi limité le nombre d'informations inscrites dans les dossiers entre autres certaines données biologiques comme le taux de troponine. Ces différentes limites peuvent aisément être dépassées moyennant la réalisation d´un registre prospectif national sur les EP en Tunisie.

## Conclusion

Le pronostic de l´EP est gouverné par la tolérance hémodynamique et le terrain sous-jacent [1]. La codification de la prise en charge de cette affection a permis une meilleure prise en charge de ces patients en fonction de leurs risques. Les facteurs prédictifs indépendants de décès et de MACE en intra-hospitalier dans notre travail étaient l'insuffisance rénale et l'appartenance au groupe HR. Un suivi à court et moyen terme relève deux fois plus de décès dans le groupe HR avec une corrélation de l´ISEP score avec la mortalité à 18 mois. L´ISEP score est corrélé à la sévérité de l´EP. Ce score n´incluant que des variables cliniques pourrait éventuellement être re-calibré en tenant compte de certaines variables biologiques. Ces résultats devraient encourager la généralisation de l´utilisation de scores de risque dans la stratification initiale des patients admis pour EP.

### Etat des connaissances sur le sujet

- *L´EP est une pathologie à l´origine d´une forte morbi-mortalité;*
- *Plusieurs scores pronostiques de l´EP ont été développés au cours des dernières années;*
- *Ces scores pronostiques ont été proposés dans le but de stratifier le risque en vue d´orienter l´approche thérapeutique qu´il s´agisse d´un traitement ambulatoire ou d´un traitement plus interventionnel*.

### Contribution de notre étude à la connaissance

- *Établir le profil épidémiologique et clinique des patientes admis pour EP dans un centre tunisien de cardiologie de niveau III;*
- *Relever la corrélation entre l´ISEP score et la gravité de l´EP;*
